# Lysophosphatidic acid receptor 4 signaling potentially modulates malignant behavior in human head and neck squamous cell carcinoma cells

**DOI:** 10.3892/ijo.2013.1849

**Published:** 2013-03-05

**Authors:** SEN MATAYOSHI, SHUNMEI CHIBA, YANFUI LIN, KAZUNARI ARAKAKI, HIROFUMI MATSUMOTO, TAKAYA NAKANISHI, MIKIO SUZUKI, SEIYA KATO

**Affiliations:** 1 Departments of Pathology and Cell Biology, Graduate School of Medicine, University of the Ryukyus, Nishihara, Okinawa 903-0215, Japan; 2 Otorhinolaryngology, Head and Neck Surgery, Graduate School of Medicine, University of the Ryukyus, Nishihara, Okinawa 903-0215, Japan

**Keywords:** lysophosphatidic acid receptor 4, head and neck squamous cell carcinoma, Rac, Rho, adenovirus vector

## Abstract

Head and neck squamous cell carcinoma (HNSCC) is the sixth most common non-skin cancer worldwide. Despite improvement in therapeutic strategies, the prognosis of advanced HNSCC remains poor. The extacellular lipid mediators known as lysophosphatidic acids (LPAs) have been implicated in tumorigenesis of HNSCC. LPAs activate G-protein-coupled receptors not only in the endothelial differentiation gene (Edg) family (LPA1, LPA2, LPA3) but also in the phylogenetically distant non-Edg family (LPA4, LPA5, LPA6). The distinct roles of these receptor isoforms in HNSCC tumorigenesis have not been clarified. In the present study, we investigated the effect of ectopic expression of LPA4 in SQ-20B, an HNSCC cell line, expressing a trivial level of endogenous LPA4. LPA (18:1) stimulated proliferation of SQ-20B cells, but did not affect proliferation of HEp-2, an SCC cell line expressing higher levels of LPA4, comparable to those of with LPA1. LPA-stimulated proliferation of SQ-20B cells was attenuated by Ki16425 and Rac1 inhibitor, but not by Y-27632. Infection with doxycycline-regulatable adenovirus vector expressing green fluorescent protein-tagged LPA4 (AdvLPA4G) abolished LPA-stimulated proliferation in SQ-20B cells with the accumulation of G2/M-phasic cells. Ectopic LPA4 induction further downregulated proliferation of Ki16425-treated SQ-20B cells, of which downregulation was partially recovered by LPA. Ectopic LPA4 induction also downregulated proliferation of Rac1 inhibitor-treated SQ-20B cells, however, LPA no longer recovered it. Finally, LPA-induced cell motility was suppressed by ectopic LPA4 expression as well as by Ki16425, Rac1 inhibitor or Y-27632. Our data suggest that LPA4 signaling potentially modulates malignant behavior of SQ-20B cells. LPA signaling, which is mediated by both Edg and non-Edg receptors, may be a determinant of malignant behavior of HNSCC and could therefore be a promising therapeutic target.

## Introduction

The annual incidence of head and neck squamous cell carcinoma (HNSCC), the sixth most common non-skin cancer in the world, is estimated to be >600,000 cases worldwide and the estimated number of deaths per year due to HNSCC is ∼350,000 (1,2). Despite improvements in therapeutic strategies including surgery, radiotherapy and/or chemotherapy, the prognosis for patients with advanced-stage HNSCC remains poor, especially owing to loco-regional recurrence (2,3). Tobacco use, alcohol consumption and human papilloma virus infection are recognized as major risk factors (2). Genetic mutation analysis data indicate that the mutational profile of HNSCC is generally consistent with those of other tumors with similar risk factors; in addition, 30% of cases harbor mutations in genes related to squamous differentiation (for example, NOTCH1, IRF6 and TP63) (4). The deregulation of specific signaling cascades such as epidermal growth factor receptor (EGFR), Ras and Wnt/β-catenin signaling have also been reported in HNSCC tumorigenesis (2,3,5). Although several molecular targeting regimens such as cetuximab (an EGFR inhibitor) and bevacizumab (a vascular endothelial growth factor receptor inhibitor) have been developed, their clinical trials have had limited efficacy and unexpected toxicities have been reported; these outcomes have emphasized the difficulties in controlling HNSCC (2,3). Further study is needed to understand the fundamental molecular basis of HNSCC tumorigenesis.

One molecule that has been implicated in HNSCC tumorigenesis is lysophosphatidic acid (LPA). LPAs are not only membrane phospholipid metabolates consisting of both saturated and unsaturated fatty acid chains but also extacellular lipid mediators that activate specific G-protein-coupled receptors (GPCRs). LPAs are ubiquitous bioactive molecules regulating various cellular events such as proliferation, migration and anti-apoptotic effects in various kinds of cells; they are thus widely involved in development, homeostatic regulations and disease processes (6–8). LPAs are produced through the hydrolysis of lysophosphatidylcholine (LPC) by autotaxin (ATX), which was initially discovered as a tumor cell motility factor which exerts extracellular lysophospholipase D activity (6–9). LPA can be also produced through the hydrolysis of phosphatidic acid by soluble phospholipase A2 (6–8), but it has been shown using ATX heterozygous knockout mice that ATX is responsible for the bulk of LPA production in serum (10). Cancer cells of several types secrete large amounts of LPC, whereupon recombinant ATX stimulates proliferation and cell motility (9). In addition, overexpression of ATX has been reported in various malignant tumors such as small cell lung carcinoma, breast cancer and Hodgkin lymphoma (6–8). Upregulated LPA production by ATX in the cancer microenvironment has been implicated in malignant behavior of tumor cells. Thus, the ATX-LPA axis is thought to be a promising target for pharmacological intervention (6–8).

LPAs bind and activate GPCRs in the endothelial differentiation gene (Edg) family (LPA1/Edg2, LPA2/Edg4, LPA3/Edg7) as well as the phylogenetically distant non-Edg family (LPA4/p2y9/GPR23, LPA5/GPR92/GPR93, LPA6/p2y5) (11–13). The Edg-family LPA receptors bind to LPAs in a similar manner and activate intracellular signaling pathways via Gi, Gq and G_12/13_ proteins, which are supposed to be responsible for the major tumorigenic processes mediated by the ATX-LPA axis (11–13). The biological role of the more recently discovered non-Edg-family receptors is not yet fully understood. LPA4 (p2y9/GPR23) was identified through the ligand screening of orphan GPCRs sharing high amino acid sequence homology with the human platelet activating factor receptor, a known GPCR (14). LPA activates G_12/13_- and Rho-mediated signaling in LPA4-expressing B103 neuroblastoma cells, which leads to neurite retraction and stress fiber formation (15,16). LPA4 signaling also evokes intracellular cAMP accumulation via Gs activation and calcium ion mobilization via Gq and Gi activation (15,16). Notably, Gs activation has not been reported downstream of the classic Edg-family LPA receptors (11–13,15,16). LPA4-deficient mice, such as LPA1- and LPA2-deficient mice, display no apparent phenotypic abnormalities, implicating the redundancy of signaling of LPA receptors (17). It has also been reported that LPA1- and LPA4-mediated signaling interact in the osteoblastic differentiation of human mesenchymal stem cells (18). LPA4 signaling also attenuates LPA1-mediated migration and invasion of B103 neuroblastoma and DLD1 colon cancer cells, suggesting functional antagonism between these two LPA receptors (17). Collectively, the expression profiles of LPA receptors and their downstream signalings are assumed to be related to malignant behavior of cancer cells, though this link has not been fully investigated.

It has been reported that LPA stimulates proliferation and motility in HNSCC cells (19). EGFR signaling has been shown to play a central role in HNSCC biology, which can be trans-activated by other receptor-mediated signaling cascades such as platelet-derived growth factor, insulin-like growth factor and LPA (2,3,5,19). However, LPA also inhibits EGF-induced activation of signal transducer and activator of transcription 1 (STAT1) in A431 esophageal squamous cell carcinoma cells (20). Thus not all the effects of LPAs can be explained by trans-activation of EGFR. In the present study, we hypothesized that LPA signaling mediated by both Edg- and non-Edg receptor family members regulates malignant behavior of HNSCC cells. Overexpression of LPA4 was attempted in SQ-20B HNSCC cells, which natively express trivial levels of LPA4. LPAs, GPCR ligands that are abundantly present in the serum and body fluids, may play an important role in the establishment of the cancer micro-environment and in the regulation of malignant behavior of HNSCC (21).

## Materials and methods

### Cell culture and reagents

HEp-2, a human squamous cell carcinoma cell line, was obtained as previously described (22). The SQ-20B cell line of laryngeal squamous cell carcinoma was kindly provided by Professor Hideyuki J. Majima (Kagoshima University Graduate School of Medical and Dental Sciences) (23). Cells were maintained in Dulbecco’s modified Eagle’s medium (DMEM) (Sigma-Aldrich, St. Louis, MO) containing 10% fetal bovine serum (FBS) (Nichirei Bioscience, Tokyo, Japan) together with antibiotics (100 U/ml penicillin and 100 *μ*g/ml streptomycin; Life Technologies, Carlsbad, CA) at 37°C in a humidified atmosphere of 5% CO_2_ and passaged with trypsin-EDTA (Life Technologies). LPA (oleoyl-l-α-LPA, 18:1) and mouse monoclonal anti-β-actin antibody (AC-15) were purchased from Sigma-Aldrich, and the goat polyclonal anti-LPA4 antibody (S-15) from Santa Cruz Biotechnology (Santa Cruz, CA). Ki16425 was purchased from Cayman Chemical Co. (Ann Arbor, Michigan MI). Rac1 inhibitor and Y-27632 were purchased from Wako (Osaka, Japan), AG1478 from Merck (Darmstadt, Germany), and doxycycline (Dox) from Clontech (Mountain View, CA).

### RNA extraction, conventional and real-time polymerase chain reaction (PCR)

Total RNA was isolated with TRIzol (Life Technologies) according to the manufacturer’s instructions. Extracted RNA (1 *μ*g) was exposed to PrimeScript II reverse transcriptase (RT) (Takara, Otsu, Japan) in a total volume of 20 *μ*l. For conventional RT-PCR, complimentary DNA obtained in 1 *μ*l of RT reaction mixture was amplified using AmpliTaq Gold PCR Master Mix (Applied Biosystems, Carlsbad, CA). PCR products were run and imaged on 1% agarose gels stained with ethidium bromide. For real-time PCR, 1 *μ*l of RT reaction mixture was amplified using Fast SYBR-Green fluorescence dye and a StepOne real-time PCR system (Applied Biosystems). Amplification reactions were performed in duplicate and fluorescence curves were analyzed with the included software. All PCR results were normalized for the expression of β-actin. Primers were designed using Primer 3 software (http://frodo.wi.mit.edu/primer3/) running on a Windows computer. A primer set for β-actin (XAHR20 and XAHR17) purchased from Funakoshi (Tokyo, Japan) was used for conventional PCR. The PCR primer sets used in the present study and the experimental conditions are listed in Table I.

### Assays for proliferation and cell motility

Viable cell numbers and proliferation rates were measured by means of WST-1 [2-(4-iodophenyl)-3-(4-nitrophenyl)-5-(2,4-disulfophenyl)-2H-tetrazolium, a tetrazolim salt], assay (Roche, Indianapolis, IN) according to the manufacturer’s instructions. Briefly, cells were inoculated on a 96-well multi-titer plate at a density of 5×10^3^ cells per well. To equilibrate the cell cycle phase, the cells were cultured in serum-free media (SFM) prior to LPA stimulation. The plates were read at wavelength of 450 nm using a scanning multi-well spectrophotometer (Bio-Rad, Model 680, Hercules, CA). For the measurement of cell motility, a wound-healing assay was performed (24). Briefly, cells were seeded on each side of an Ibidi culture insert for live cell analysis (Ibidi, Munich, Germany) and the area filled with migrated cells was observed using an Olympus phase-contrast microscope (model CKX41, Tokyo, Japan) connected to a DP50 digital camera (Olympus). Image analysis was performed using Image J software (NIH, Bethesda, MD).

### Cell cycle analysis

For flow cytometric analysis of the cell-cycle distribution, cells were harvested using trypsin-EDTA and fixed with 70% ethanol. Fixed cells (1×10^5^) were stained with 200 *μ*l of Guava cell cycle reagent (Millipore, Billerica, MA) and analyzed using the Guava Personal Cell Analysis System (Millipore) according to the manufacturer’s instructions.

### Recombinant adenovirus vector

Full-length cDNA of human LPA4 with C-terminal turbo green fluorescence protein (tGFP) tag (Origene, Rockville, MD) was subcloned into the recombinant adenovirus vector (AdvLPA4G) using an Adeno-X Tet-On 3G system (Clontech) according to the manufacturer’s instructions. AD293 cells (Agilent Technologies, Santa Clara, CA) were used as a packaging cell line and a ViraBind™ adenovirus purification kit (Cell Biolabs, San Diego, CA) was used for amplification. Working stocks of viruses were stored in aliquots at −80°C. Titer was determined by means of a conventional plaque assay using Noble Agar (Difco, Detroit, MI). Dox-negative condition was used as a negative control. Transfection efficiencies were tested with GFP fluorescence as observed with an Olympus fluorescent microscope.

### Western blotting

Whole cell extracts were obtained in RIPA buffer (Santa Cruz Biotechnology) and were then subjected to the Quick Start Bradford Protein Assay kit (Bio-Rad). Whole cell extracts (30 *μ*g) were subsequently resolved in 10% sodium dodecyl sulfate (SDS)-polyacrylamide gel electrophoresis (PAGE) and were electronically transferred to a nitrocellulose membrane (Bio-Rad). The membranes were probed with a 1:200 dilution of a goat polyclonal anti-LPA4 followed by incubation with a 1:5000 dilution of peroxidase-conjugated secondary antibody-like particle (supplied in an XL-SAP kit for western blotting, APRO Life Science, Naruto, Japan). The proteins were subsequently developed using ImmunoStar LD reagents (Wako) and visualized with a luminescent imager (Ez-Capture, ATTO Co., Tokyo, Japan). Alternatively, the blots were incubated with Restore PLUS Western Blot Stripping Buffer (Thermo Fisher Scientific, Waltham, MA) and re-probed with a 1:2000 dilution of anti-β-actin antibody.

### Statistical analysis

Experimental groups were compared using analysis of variance (ANOVA) and, when appropriate, Student’s t-test. The data are expressed as the mean ± SEM. A level of p<0.05 was considered statistically significant.

## Results

### Expression of LPA1 and LPA4 in human squamous cell carcinoma cells

**Figure 1:**
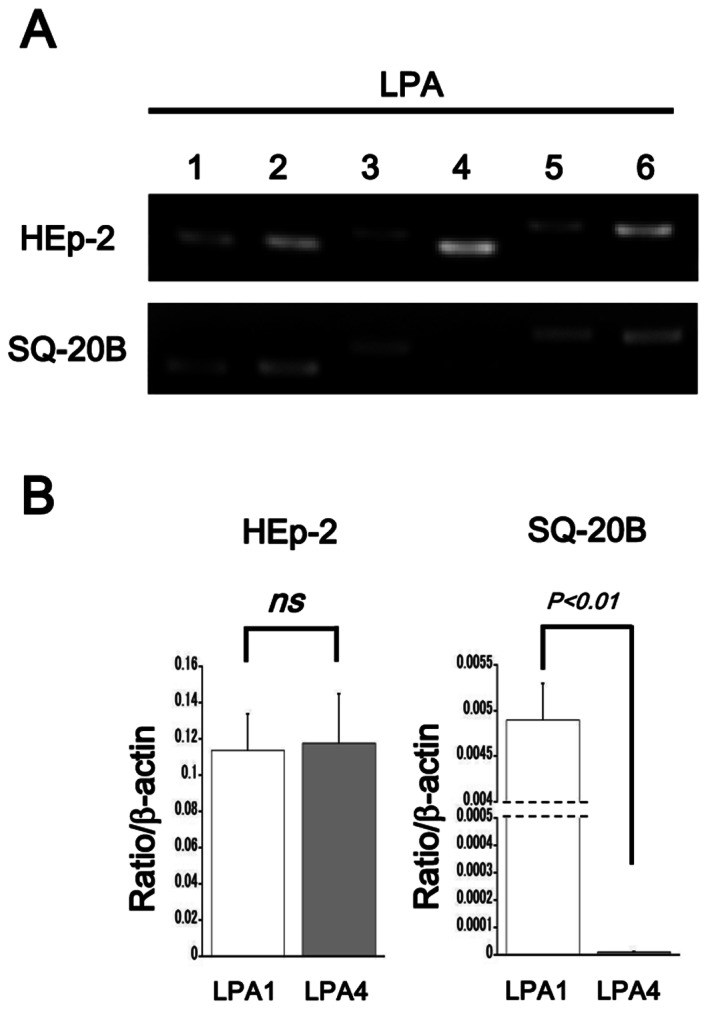
Expression profile of LPA receptors in HEp-2 and SQ-20B cells. (A) Conventional RT-PCR for LPA1-6. (B) Real-time PCR for LPA1 (open bars) and LPA4 (gray bars). Data are shown as the mean ± SEM (n=3). P-values are indicated; ns, not significant.

The expression profiles of LPA receptors in various cancer cell types were screened with conventional RT-PCR. HEp-2 cells expressed all isoforms of LPA receptors (LPA1-6, Fig. 1A, upper panel). SQ-20B cells expressed all Edg family LPA receptors (LPA1–3) and 2 isoforms of the non-Edg-family LPA receptors (LPA5 and LPA6). Slight expression of LPA4 was detected in SQ-20B cells (Fig. 1A, bottom panel). Real-time PCR revealed that expression levels of LPA1 and LPA4 were similar in HEp-2 cells, while only a trivial level of LPA4 expression was seen in SQ-20B cells (Fig. 1B).

### LPA stimulated proliferation in SQ-20B cells but not in HEp-2 cells

**Figure 2:**
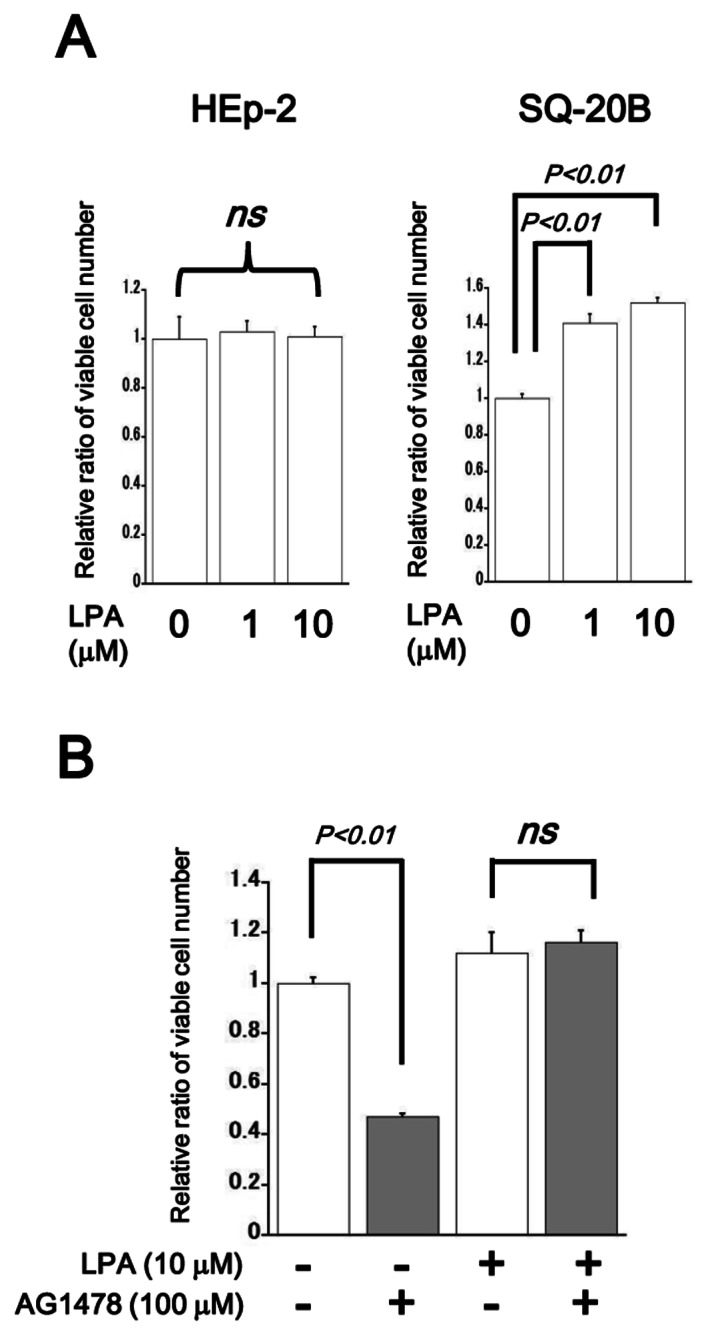
Proliferation assay of LPA-stimulated HEp-2 and SQ-20B cells. (A) Cells were starved with SFM for 24 h. WST-1 assay was performed 48 h after the LPA stimulation (concentrations are indicated). (B) SQ-20B cells were stimulated with LPA (10 *μ*M) with (gray bars) or without (open bars) AG1478 treatment. AG1478 (100 *μ*M) was added 30 min prior to LPA stimulation. WST-1 assay was performed as above. Data are shown as the mean ± SEM (n=6). P-values are indicated; ns, not significant.

In HEp-2 cells, WST-1 assay revealed no mitogenic response against LPA. In SQ-20B cells, on the other hand, LPA stimulated proliferation in a dose-dependent fashion (Fig. 2A). Thus, further experiments were performed using LPA-responsive SQ-20B cells. Since it has been reported that LPA-induced mitogenic response largely depends on the transactivation of EGFR in some HNSCC cell lines (19), we tested the effect of AG1478, a specific inhibitor for EGFR. In the absence of LPA, AG1478 reduced proliferation of SQ-20B cells, suggesting the endogenous activation of EGFR in this cell line. In the presence of LPA, however, treatment with AG1478 did not result in reduced proliferation of SQ-20B cells, suggesting that LPA signaling stimulates proliferation of SQ-20B cells independently from EGFR activation (Fig. 2B).

### LPA stimulated proliferation of SQ-20B cells via the activation of Ki16425-sensitive Edg family receptors and Rac1

**Figure 3:**
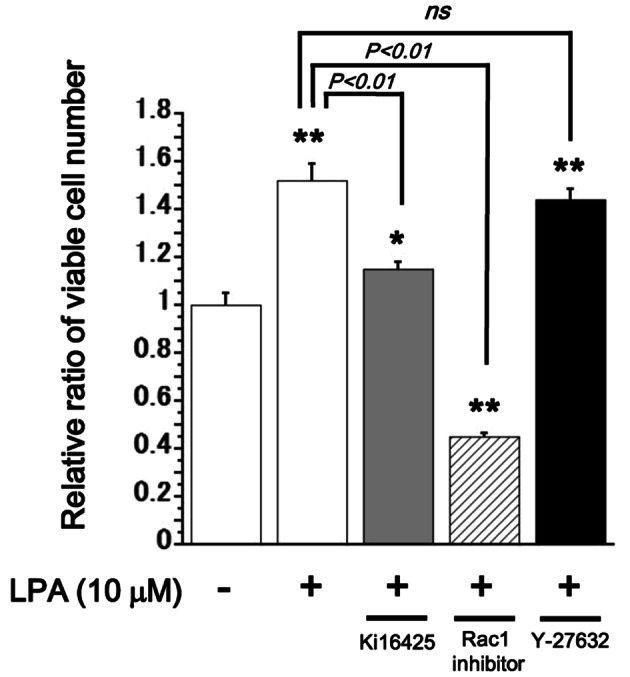
Proliferation assay of LPA-stimulated SQ-20B cells. Cells were starved with SFM for 24 h. WST-1 assay was performed 48 h after the LPA stimulation (10 *μ*M) with or without (open bars) treatment with the inhibitors. Ki16425 (gray bar, 10 *μ*M), Rac1 inhibitor (hatched bar, 50 *μ*M), or Y-27632 (filled bar, 10 *μ*M) was added 30 min prior to LPA stimulation. Data are shown as the mean ± SEM (n=6). ^*^P<0.05;^**^P<0.01 against the control LPA-unstimulated cells. P-values are indicated; ns, not significant.

To investigate the intracellular signaling mechanism responsible for LPA-stimulated proliferation in SQ-20B cells, the effects of the LPA1 and LPA3 inhibitor Ki16425 (10 *μ*M), Rac1 inhibitor (50 *μ*M) and the Rho-associated coiled-coil forming kinase (ROCK) inhibitor Y-27632 (10 *μ*M) were tested (25). Treatment with Ki16425 or a Rac1 inhibitor inhibited proliferation of LPA (10 *μ*M)-stimulated SQ-20B cell growth, whereas, treatment with Y-27632 showed no significant effect on proliferation in these cells (Fig. 3).

### Overexpression of LPA4 in SQ-20B cells

**Figure 4:**
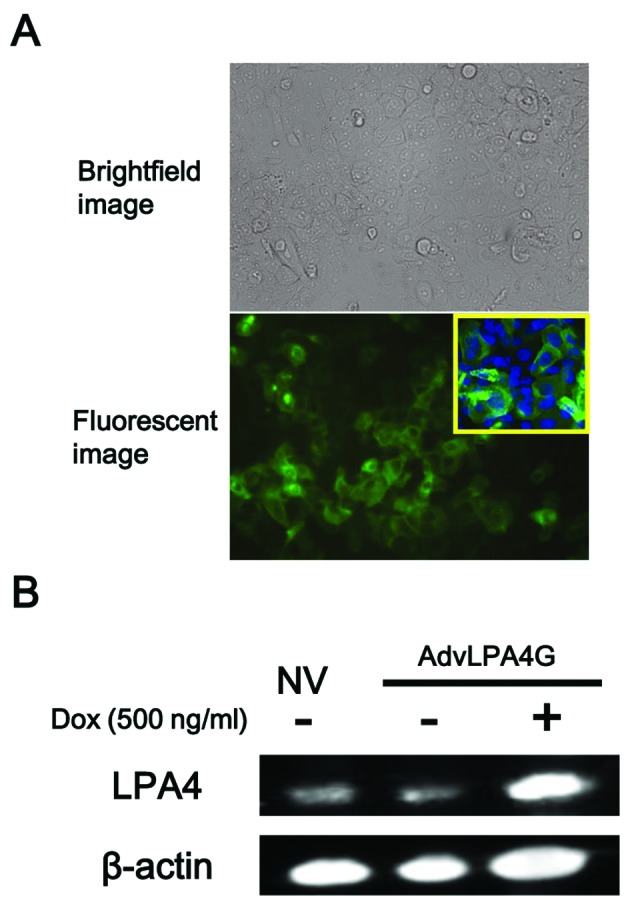
Ectopic expression of GFP-tagged LPA4 in SQ-20B cells. (A) Observation by phase-contrast microscopy (inset; high magnification). SQ-20B cells were infected with 100 MOI of AdvLPA4G. Ectopic expression of GFP-tagged LPA4 was observed 48 h after infection with Dox treatment (500 ng/ml). (B) Western blotting for LPA4 and β-actin. SQ-20B cells were infected with 100 MOI of AdvLPA4G and were then incubated with 500 ng/ml of Dox. Cell lysates were harvested 48 h after infection. NV, no virus infected control cells.

Next, we attempted overexpression of LPA4 in SQ-20B cells which indigenously exhibit a trivial level of LPA4 expression (Fig. 1). A fluorescent image showed that AdvLPA4G (100 MOI, multiplicity of infection) infected cells represented membranous and cytoplasmic expression of GFP-associated LPA4 protein in the presence of Dox (100–500 ng/ml of concentration was used in the present study) (Fig. 4A). Western blot analysis also showed upregulated expression of LPA4 in Dox-treated AdvLPA4G-infected cells (Fig. 4B).

### Overexpression of LPA4 inhibited LPA-induced mitogenic response in SQ-20B cells

**Figure 5:**
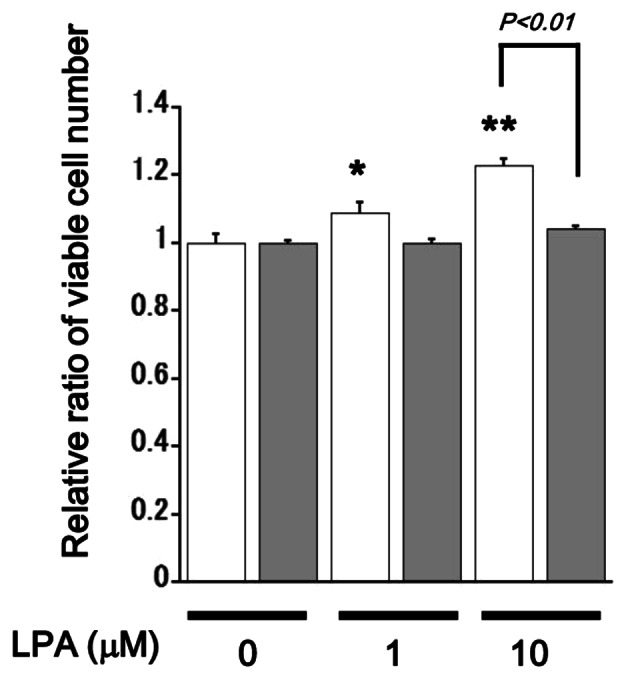
Proliferation assay of AdvLPA4G-infected SQ-20B cells. Cells were infected with 100 MOI of AdvLPA4G and were then incubated for 24 h with (gray bars) or without (open bars) 100 ng/ml of Dox in SFM. WST-1 assay was performed 48 h after the LPA stimulation (concentrations are indicated). Data are shown as the mean ± SEM (n=6). ^*^P<0.05; ^**^P<0.01 against the control unstimulated cells. P-values are indicated; ns, not significant.

**Figure 6:**
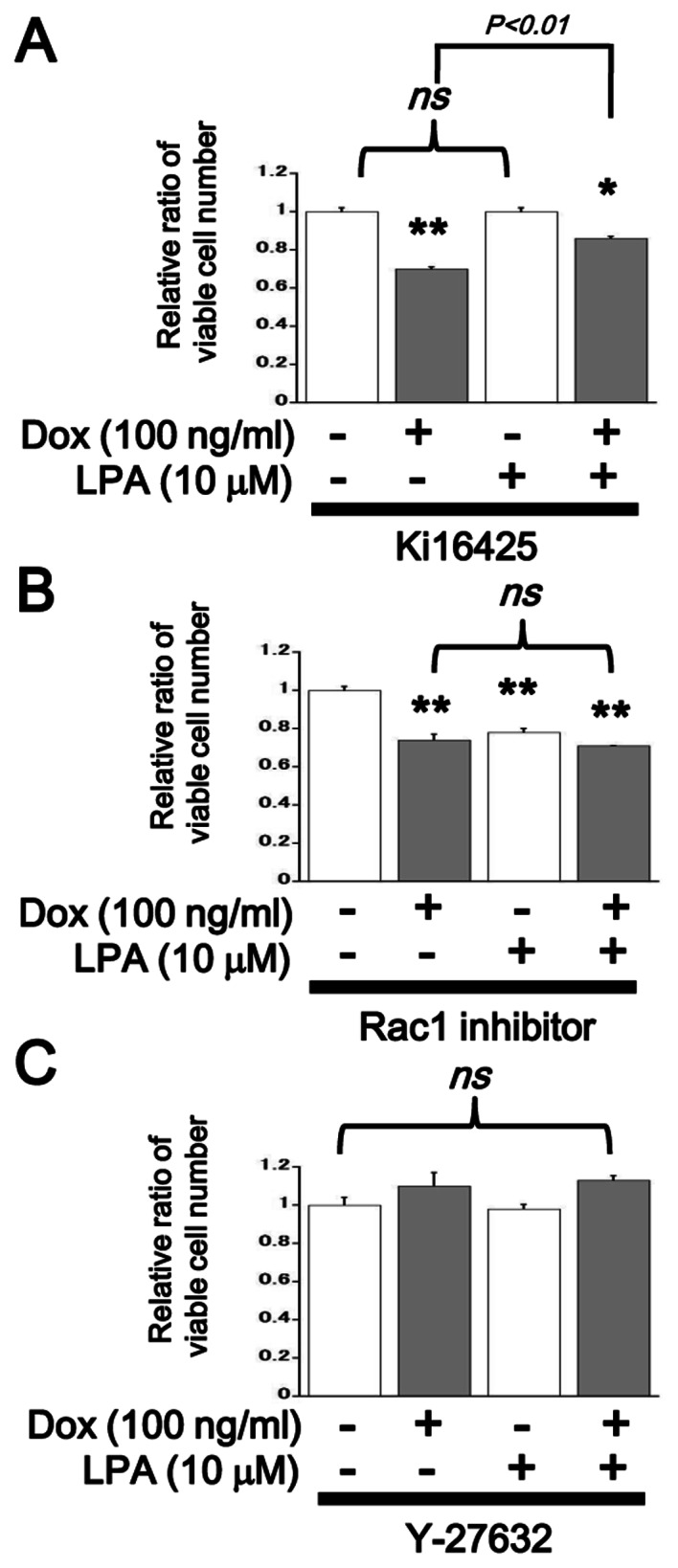
Proliferation assay of AdvLPA4G-infected SQ-20B cells in the presence of inhibitors. Cells were infected with 100 MOI of AdvLPA4G and were then incubated for 24 h with (gray bars) or without (open bars) 100 ng/ml of Dox in SFM. WST-1 assay was performed 48 h after the LPA stimulation (10 *μ*M). Ki16425 [(A) 10 *μ*M], Rac1 inhibitor [(B) 50 *μ*M], or Y-27632 [(C) 10 *μ*M] was added 30 min prior to LPA stimulation. Data are shown as the mean ± SEM (n=6). ^*^P<0.05; ^**^P<0.01 against the control unstimulated cells. P-values are indicated; ns, not significant.

LPA induced a mitogenic response in SQ-20B cells in the Dox-free control condition; this is consistent with the result shown in Fig. 2A. In ectopic LPA4-expressing cells, in contrast, LPA-induced mitogenic response was completely inhibited (Fig. 5). In the presence of Ki16425, proliferation of SQ-20B cells was attenuated by the induction of ectopic LPA4 but could be partially rescued by the addition of LPA (Fig. 6A). In the presence of Rac1 inhibitor, proliferation of SQ-20B cells was suppressed irrespective of LPA treatment and no further reduction resulted from ectopic LPA4 induction (Fig. 6B). In the presence of Y-27632, no significant change in proliferation of SQ-20B cells was observed upon LPA treatment or ectopic LPA4 induction (Fig. 6C). Flow cytometric cell cycle analysis showed that the percentage of G2/M-phasic cells was increased 6 h after LPA stimulation in ectopic LPA4-expressing SQ-20B cells (Table II).

### Inhibition of cell motility in LPA4-expressing SQ-20B cells

**Figure 7:**
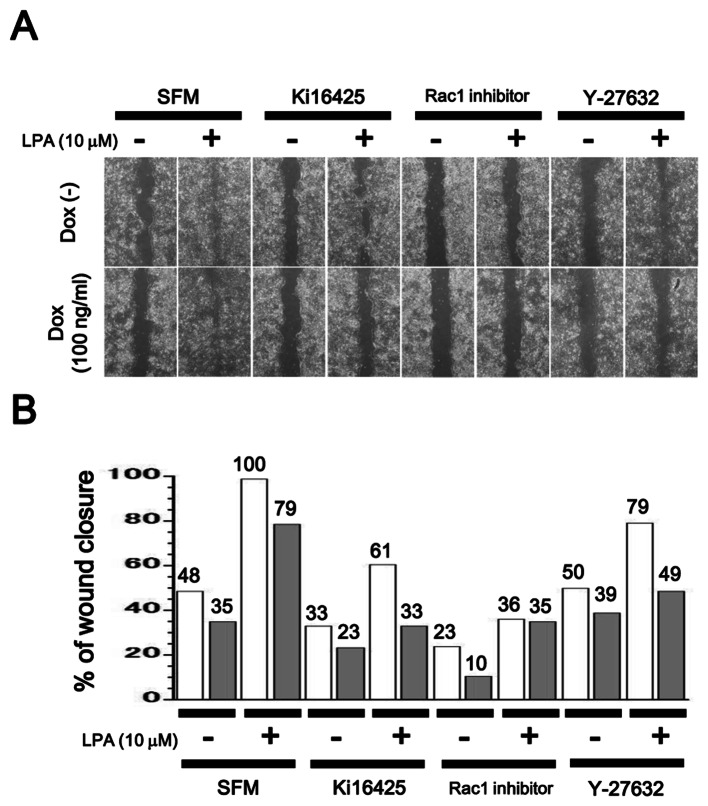
Wound healing assay of AdvLPA4G-infected SQ-20B cells. Cells were infected with 100 MOI of AdvLPA4G and incubated with [(A) lower panels; (B) gray bars] or without [(A) upper panels; (B) open bars] 100 ng/ml of Dox overnight followed by re-seeding on Ibidi culture inserts. Cells were pretreated with or without the inhibitors (Ki16425, 10 *μ*M; Rac1 inhibitor, 50 *μ*M; or Y-27632, 10 *μ*M) before removal of the insert and then stimulated with LPA (10 *μ*M) for 8 h. Percentage of wound closure (maximum 100%) in each condition was measured as described in Materials and methods (values are also indicated at the top of individual bars). Representative data of two independent experiments are shown. (A) Phase-contrast microscope images. (B) Percentage of wound closure in each condition.

Cell motility was measured through a wound healing assay. LPA upregulated cell motility in SQ-20B cells, while additional treatment with Ki16425, Rac1 inhibitor or Y-27632, suppressed it. Ectopic induction of LPA4 also reduced cell motility regardless of the presence or absence of inhibitors (Fig. 7).

## Discussion

In the present study, we demonstrated that adenovirus-mediated ectopic induction of LPA4 signaling potentially modulates malignant behavior of SQ-20B, HNSCC cells including proliferation (Figs. 5 and 6) and cellular motility (Fig. 7). Activation of Ki16425-sensitive Edg receptrors (LPA1 and LPA3) and Rac1 was identified as an important mitogenic cascade with which LPA4 signaling may interfere (Figs. 3 and 6). Signaling mediated by both Edg and non-Edg receptors may be a determinant of malignant behavior of HNSCC and may therefore be a promising therapeutic target.

It is known that Edg-family LPA receptors (LPA1, LPA2, LPA3) have a ubiquitous distribution in most tissues. Non-Edg LPA receptors, on the other hand, appear to have low expression levels in many tissues (11–13). Exceptionally, high levels of LPA4 expression have been observed in the ovaries (11–14) and LPA5 expression has been identified in the small intestine, spleen and dorsal root ganglion cells (11–13,26). LPA6 expression has been shown in the hair follicles and vascular endothelium (11,27). In various other types of cells and tissues, however, the expression profiles of LPA receptors have not been investigated in detail. Here, we found that LPA4 was expressed at different levels in two different SCC cell lines, HEp-2 and SQ-20B (Fig. 1). More importantly, LPA stimulated proliferation only in SQ-20B cells, which exhibit trivial levels of LPA4 expression (Fig. 2). In our preliminary experiments, Detroit-562, another HNSCC cell line and HCT116, a colorectal cancer cell line, showed mild mitogenic responses against LPA in accordance with low levels of LPA4 expression (data not shown).

It was also previously reported that LPA-induced malignant behavior of cancer cells are largely dependent on Edg-family receptor activation (6–8,11–13,21). Consistently, we observed the inhibition of LPA-induced mitogenic response in SQ-20B cells by Ki16425, an inhibitor for LPA1 and LPA3 (Fig. 3). On the contrary, LPA4, a non-Edg LPA receptor, potentially acts as a negative regulator for certain cellular events mediated by Edg-family receptors: for example, during osteoblast differentiation, LPA1 and LPA4 have been shown to exert distinct functions (18). Unfortunately, no specific inhibitor for LPA4 is available to date (12), but the development of one would be highly beneficial for shedding light on the role of LPA4 in physiological and disease processes.

Rho-family GTPases including Rho, Rac and Cdc42 are presumed to modulate various cellular functions such as cytoskeletal reorganization, cell motility, invasion, proliferation and apoptotic processes (28,29). Rho-family GTPases are also major intracellular signaling molecules downstream of GPCRs including the LPA receptors (11–13). The mitogenic effect of LPA on SQ-20B cells was attenuated by Ki16425 and Rac1 inhibitor. Thus, the Gi-Rac signaling axis may play a role in LPA-induced proliferation downstream of Ki16425-sensitive Edg receptors (LPA1 and LPA3) (11–13). Y-27632, a Rho/ROCK inhibitor, had no significant effect on the proliferation of LPA-stimulated SQ-20B cells (Fig. 3). Among known LPA receptors, LPA4 has been shown to bind only to G_12/13_ proteins and to activate Rho (11–13). However, the G_12/13_-Rho/ROCK pathway is not expected to be involved in the regulation of proliferation in LPA-stimulated SQ-20B cells.

Ectopic induction of LPA4 abolished LPA-induced mitogenic response in SQ-20B cells (Fig. 5), suggesting that LPA4 signaling acts as a negative regulator for proliferation. In the presence of Ki16425, LPA mildly recovered cell proliferation of ectopic LPA4 expressing SQ-20B cells (Fig. 6A), probably due to partial release from competitive inhibition by Ki16425 against LPA1 and LPA3 (30). In the presence of Rac1 inhibitor, ectopic expression of LPA4 no longer suppressed proliferation of SQ-20B cells (Fig. 6B). We also observed inhibition of LPA-induced Rac1 activation in ectopic LPA4 expressing SQ-20B cells using a pull-down assay (data not shown). Thus, LPA4 signaling may interfere with Rac1 activation in LPA-stimulated SQ-20B cells.

In our flow cytometric analysis, LPA-stimulated SQ-20B cells showed an accumulation of G2/M-phasic cells with an ectopic induction of LPA4 (Table II). Similarly, Rat-2, a rat fibroblast cell line, expressing dominant negative Rac1 (N17rac1) has been shown consistently to exhibit growth arrest at the G2/M phase (31). In the presence of Y-27632, a Rho/ROCK inhibitor, no significant changes were seen in the proliferation of SQ-20B cells irrespective of LPA4 induction (Fig. 6C). In various systems, it has been indicated that the activities of Rac and Rho may be antagonistic through their regulation of GEF (guanine nucleotide exchange factors, which act as activators) and GAP (GTPase-activating proteins, which act as inhibitors) (29,32–34). In the present study, however, we could not confirm the involvement of the G_12/13_-Rho/ROCK pathway in the regulation of SQ-20B cell proliferation downstream of LPA4. Further study is needed to identify the LPA4-mediated inhibitory pathway involved in the LPA-induced and Gi/Rac-mediated mitogenic response in these cells.

LPA stimulates not only proliferation but also cell motility in HNSCC cells (19,35,36). Therefore, we examined the role of LPA4 signaling on cell motility in SQ-20B cells. Our wound healing assay data suggested that LPA-induced cell motility is mediated by Ki16425-sensitive Edg family receptor activation and the exogenously induced LPA4 signaling negatively regulates cell motility in SQ-20B cells (Fig. 7). Given that the inhibitor for either Rac1 or Rho/ROCK attenuated cell motility, these small G-proteins must play an important role in promoting cell motility in these cells. Rho proteins induce stress fiber and focal adhesion contact formation, whereas Rac and Cdc42 are involved in the formation of lamellipodia and filopodia (28,29,32–34). Irrespective of any antagonistic relationship between the Rac and Rho/ROCK pathways (32–34), these small G-proteins would coordinately promote changes in cell motility (37). It has been reported that cell motility induced by LPA is associated with activation of RhoA and inhibition of Akt and Rac in embryonic fibroblasts derived from LPA4-deficient mice (17). It has also been reported that ATX promotes invasion in HT1080, fibrosarcoma cells via the activation of cyclic AMP/EPAC (exchange protein directly activated by the cyclic AMP)/Rac1 pathway at the downstream of LPA4 (38). Our data suggest that LPA4 signaling negatively modulates cell motility in HNSCC. The regulatory mechanism involved in this process, including the Rac and Rho/ROCK pathways, should be clarified in further investigations.

Known LPA receptors (LPA1–6) have been shown to mediate major cellular events through their effects on LPAs, though some LPA-mediated cellular functions may be mediated by the intracellular signaling molecule peroxisome proliferator-activated receptor (PPARγ) (39,40). Moreover, 2,3-cyclic phosphatidic acid, an endogenously produced PPARγ antagonist, that is similar in structure to LPA, inhibits cancer cell invasion and metastasis *in vitro* and *in vivo* (41). In IMR-32 human neuroblastoma cells, however, LPA antagonizes 15-deoxy-Δ12,14-prostaglandin J2-mediated PPARγ activation (42). Although we did not test the activation level of PPARγ in LPA-stimulated SQ-20B cells, the possibility of an interaction between trans-membrane LPA receptors and the intracellular targets of LPA in HNSCC needs to be addressed in a future study.

## Figures and Tables

**Table I:** Sequences of primers and experimental conditions for PCR.

A, Primers for the conventional PCR				
Genes	Sense primer (5-3′)	Antisense primer (5-3′)	Annealing temperature (°C)	Amplified size (bp)

LPA1	cgtgctggcctatgagaaat	tgtgaactccagccaagatg	60	209
LPA2	ctgctcctggatggtttagg	tgggcagaggatgtatagtgg	60	209
LPA3	ggacacccatgaagctaat	tctgggttctcctgagagaa	60	256
LPA4	ctcttcgcaagcctgctact	gttcagagttgcaaggcaca	60	221
LPA5	tctcccgtgtcctgactacc	gccgtacatgttcatctgga	60	286
LPA6	cagaagccacatggaaaaca	tgctgccactactgagcaat	60	287
β-actin	acccacactgtgcccatcta	cggaaccgctcattgcc		
	(XAHR20 primer)	(XAHR17 primer)	60	289

B, Primers for the real-time PCR				
Genes	Sense primer (5-3′)	Antisense primer (5-3′)	Annealing temperature (°C)	Amplified size (bp)

LPA1	tgcttggggcctttatcatc	ttctcataggccagcacgtc	60	94
LPA2	atcatcctgggggcgttc	cattgcaggactcacagccta	60	85
LPA3	taggggcgtttgtggtatgc	caccttttcacatgctgcac	60	97
LPA4	ccatgggtgacagaagattca	ggcagtagcattgcccaac	60	83
LPA5	tctctgctgctgatgaagctg	agggaggtcatgggaatgtg	60	92
LPA6	ccagcggaaattttacagca	gcaaattatctggatctttggatg	60	99
β-actin	atccgcaaagacctgtacgc	ccagggcagtgatctccttc	60	97

**Table II:** Flow cytometric cell cycle analysis.

	G0/G1	S	G2/M
Dox(−) control	52.8±1.14	11.4±0.33	35.6±0.78
Dox(+)	38.2±0.44^a^	9.83±0.15^a^	51.9±0.22^a^

SQ-20B cells were starved in SFM for 24 h and flow cytometric cell cycle analysis was performed 6 h after LPA (10 *μ*M) stimulation. Percentages of cells in each cell cycle phase are shown as mean ± SEM.

a P<0.01 against the controls.
